# Supplementary material for: Novel Driver Strength Index highlights important cancer genes in TCGA PanCanAtlas patients
Source: PeerJ. 2022 Aug 11;10:e13860. doi: 10.7717/peerj.13860 (PMC9375969; doi:10.7717/peerj.13860)
Supplement: Supplemental Information 2 [file peerj-10-13860-s002.zip › COHORTS/genes plots/age/2021_8_16_14_9_distribution_cohorts_males_45-49.pdf]

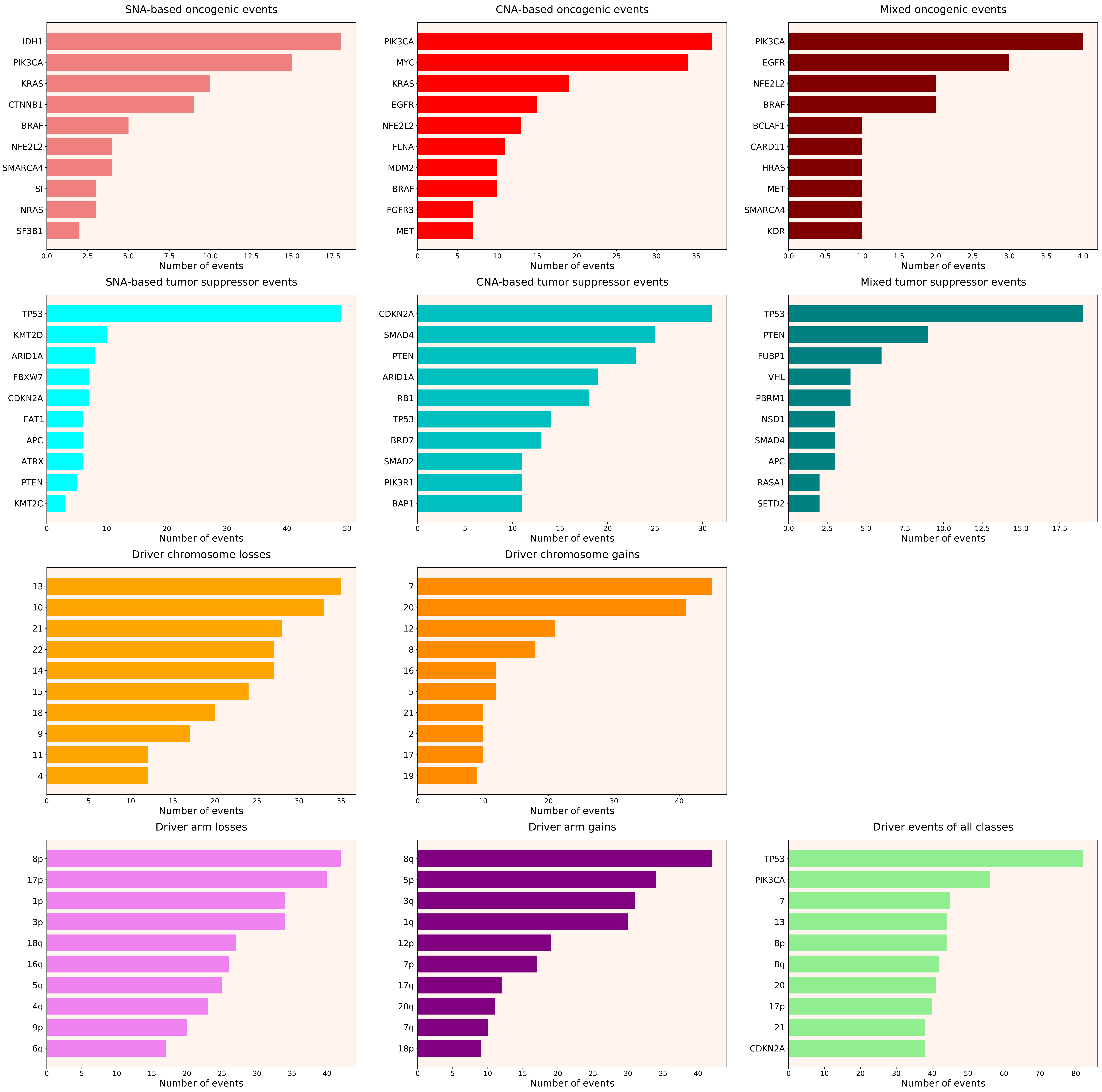

SNA-based oncogenic events
IDH1
PIK3CA
KRAS
CTNNB1
BRAF
NFE2L2
SMARCA4
SI
NRAS
SF3B1
0.0 2.5 5.0 7.5 10.0 12.5 15.0 17.5
Number of events
CNA-based oncogenic events
PIK3CA
MYC
KRAS
EGFR
NFE2L2
FLNA
MDM2
BRAF
FGFR3
MET
0 5 10 15 20 25 30 35
Number of events
Mixed oncogenic events
PIK3CA
EGFR
NFE2L2
BRAF
BCLAF1
CARD11
HRAS
MET
SMARCA4
KDR
0.0 0.5 1.0 1.5 2.0 2.5 3.0 3.5 4.0
Number of events
SNA-based tumor suppressor events
TP53
KMT2D
ARID1A
FBXW7
CDKN2A
FAT1
APC
ATRX
PTEN
KMT2C
0 10 20 30 40 50
Number of events
CNA-based tumor suppressor events
CDKN2A
SMAD4
PTEN
ARID1A
RB1
TP53
BRD7
SMAD2
PIK3R1
BAP1
0 5 10 15 20 25 30
Number of events
Mixed tumor suppressor events
TP53
PTEN
FUBP1
VHL
PBRM1
NSD1
SMAD4
APC
RASA1
SETD2
0.0 2.5 5.0 7.5 10.0 12.5 15.0 17.5
Number of events
Driver chromosome losses
13
10
21
22
14
15
18
9
11
4
0 5 10 15 20 25 30 35
Number of events
Driver chromosome gains
7
20
12
8
16
5
21
2
17
19
0 10 20 30 40
Number of events
Driver arm losses
8p
17p
1p
3p
18q
16q
5q
4q
9p
6q
0 5 10 15 20 25 30 35 40
Number of events
Driver arm gains
8q
5p
3q
1q
12p
7p
17q
20q
7q
18p
0 5 10 15 20 25 30 35 40
Number of events
Driver events of all classes
TP53
PIK3CA
7
13
8p
8q
20
17p
21
CDKN2A
0 10 20 30 40 50 60 70 80
Number of events
